# Novel insights into the post-translational modifications of Ydj1/DNAJA1 co-chaperones

**DOI:** 10.1016/j.cstres.2023.11.001

**Published:** 2023-11-17

**Authors:** Megan M. Mitchem, Courtney Shrader, Elizabeth Abedi, Andrew W. Truman

**Affiliations:** Department of Biological Sciences, The University of North Carolina at Charlotte, Charlotte, NC 28223, USA

**Keywords:** Ydj1, DNAJA1, PTMs, Co-chaperones, Chaperone code

## Abstract

The activity of the Hsp70 molecular chaperone is regulated by a suite of helper co-chaperones that include J-proteins. Studies on J-proteins have historically focused on their expression, localization, and activation of Hsp70. There is growing evidence that the post-translational modifications (PTMs) of chaperones (the chaperone code) fine-tune chaperone function. This mini-review summarizes the current understanding of the role and regulation of PTMs on the major J-proteins Ydj1 and DNAJA1. Understanding these PTMs may provide novel therapeutic avenues for targeting chaperone activity in cancer and neurodegenerative diseases.

## Introduction

Protein folding is critical for cell viability, and mechanisms that promote protein folding (proteostasis) are well-conserved throughout all organisms. Heat shock proteins (HSPs) are intimately involved in this process, binding nascent polypeptide chains and aiding in folding as well as refolding denatured proteins.^1^ Molecular chaperones do not act alone in this process; they bind to a suite of “co-chaperone” helper proteins that fine-tune their specificity and activity. The J-proteins are one of the most robustly studied groups of co-chaperones.^2^ The first J-protein was identified in *Escherichia coli* and was subsequently named DnaJ due to its role in DNA replication.^3^, ^4^ Given the essential nature of molecular chaperones, DnaJ-like proteins are found in all organisms, from bacteria to humans.^1^ One of the most well-studied J-proteins, Ydj1, is present in the budding yeast *Saccharomyces cerevisiae*. This gene was identified from a yeast expression library using antisera generated against the matrix laminar pore complex.^5^ In this study, Ydj1 was identified as being present in both soluble and microsomal fractions and required for yeast high-temperature growth.^5^ The human equivalent of Ydj1, DNAJA1, was isolated from a human umbilical vein endothelial cDNA library.^6^ The human homolog shows 41% identity to *E. coli* DnaJ, with the highest degree of homology in the J-domain required for interaction with Hsp70.^6^ DNAJA1 and Ydj1 aid in protein folding by binding to newly synthesized substrates (clients) and unfolded proteins. They transport the bound client to Hsp70, where the client forms a transient complex with the open peptide binding sites of Hsp70. This process stimulates the Hsp70 ATPase, causing the lid of Hsp70 to close, stabilizing the client interaction and promoting protein folding^2^.

## Cellular roles for DNAJA1/Ydj1

### Response to thermal stress

Cells continually encounter proteotoxic stresses such as heat shock and must respond readily to maintain viability. Mammalian and yeast heat shock elements facilitate the expression of specialized genes involved in proteostasis and form a tightly-knit group centered around Hsp70, 40, and Hsp90.^7^ Upon loss of Ydj1, cells become incredibly sensitive to heat stress – indicating its vital role in combating thermal dysregulation.^8^ This may be partially due to the fact that Ydj1 stabilizes several proteins, including Pkc1, that are instrumental in maintaining cell wall integrity at high temperatures.^9^ Upon acute heat stress to cells, proteins form reversible quinary complexes that protect key cellular machinery.^10^, ^11^, ^12^ Although Ydj1 is critical for the disaggregation of many proteins, including the model substrate luciferase, it is dispensable for the resolution of heat-induced Pab1 condensates^10^.

### Response to replicative stress

Recent studies have shown that Ydj1 regulates the activity of ribonucleotide reductase (RNR), a key enzyme in DNA synthesis. Ydj1 binds and stabilizes Rnr2, a small subunit of the RNR complex.^13^ Similarly, DNAJA1 interacts similarly with the human version of Rnr2 (R2B) and regulates the RNR stability in human cells. Importantly, inhibition of DNAJA1 with the small molecule 116–9e promotes R2B degradation and sensitizes cancer cells to RNR inhibitors such as hydroxyurea and triapine.^13^ Overall, this and subsequent studies set the stage for novel anticancer inhibitors based on disruption of the chaperone-RNR complex.

### Cancer

Several studies have suggested that DNAJA1 inhibition may provide a novel anticancer strategy. Castration-resistant prostate cancer cells (CRPCs) overexpressing the androgen receptor are insensitive to Hsp90 inhibitors but are sensitive to Hsp40 inhibition.^14^ A chemogenomic screen comparing the small molecule resistance of wild-type HAP1 cells to those lacking DNAJA1 revealed a pleiotropic role for DNAJA1 in cancer.^15^ Although the loss of DNAJA1 sensitized the mammalian haploid cell line HAP1 to 31% of the drugs screened, DNAJA1 knockout cells were more resistant to 14% of the compounds.^15^ While speculative at this time, these data may suggest that DNAJA1 mutations in patients may play a role in resistance to prescribed anticancer therapies.

### Neurodegenerative diseases

DNAJA1 has been implicated in various diseases, particularly neurodegenerative disorders characterized by tau aggregation and amyloidogenesis.^16^ The interaction between Hsp70 and the tau-DNAJA1 complex prevents tau degradation, suggesting that blocking the binding of DNAJA1 to Hsp70 could redirect tau towards a clearance pathway. Other studies have also implicated DNAJA1 in the pathogenesis of other proteins involved in neurodegenerative disorders, such as amyloid beta (Abeta).^17^ In Alzheimer’s disease (AD), the accumulation of amyloid beta (Abeta) peptides throughout the cell triggers neurotoxic events, including mitochondrial dysfunction. It has been hypothesized that in a normal cell, Ydj1/DNAJA1 assists in degrading aggregation-prone Abeta by translocating it to the mitochondria. However, when there is a malfunction in this machinery, this pathway can become maladaptive.^16^ Ydj1/DNAJA1 counterintuitively promotes the stabilization of the toxic Abeta aggregates instead of preventing them and regulating their transport to the mitochondria. More effective treatment modes may be elucidated by further understanding the mechanisms behind these diseases and determining ways to fine-tune the cellular response^18^.

## J-protein classifications and families

**Fig. 1:**
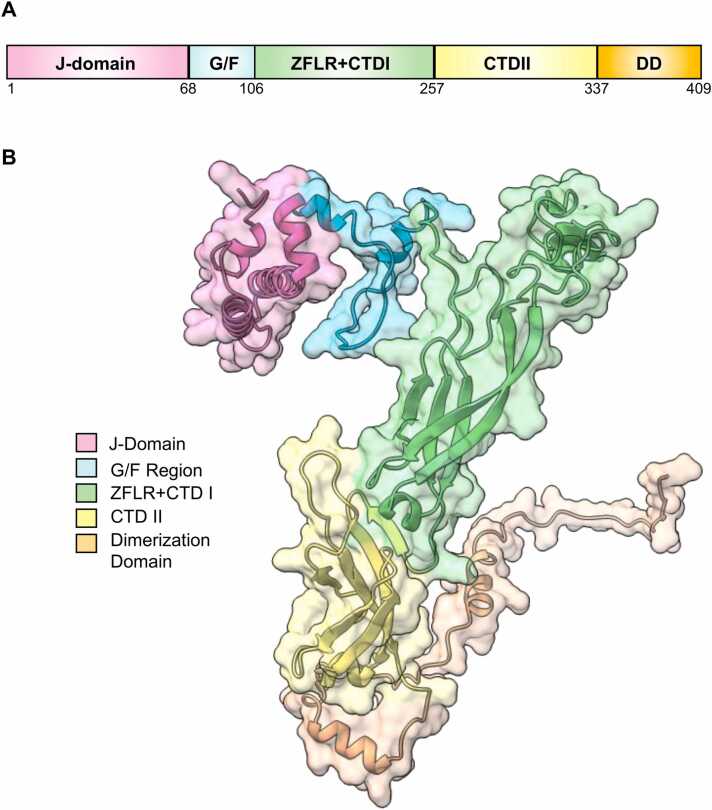
Domains of yeast Ydj1. (A) Domains of Ydj1. J-domain, G/F region: glycine and phenylalanine-rich region. The ZFLR: zinc finger-like region. The CTDI: carboxyl-terminal domain I. The CTDII: carboxyl-terminal domain II. The DD: dimerization domain. (B) Predicted structure of a full-length Ydj1 protomer (Alphafold A0A816B2×6).

Ydj1 and DNAJA1 are Hsp40s, also referred to as J-proteins, a group of proteins containing a conserved region of around 70 amino acids in the J-domain.^2^ J-domain proteins have been sorted into three classes: I, II, and III. **Class I** is categorized based on the motifs and domains present in *E. coli* DnaJ. Their domains are as follows: a J-domain, a glycine/phenylalanine-rich region, a zinc finger-like region, a carboxyl-terminal domain (CTD) I domain, CTD II domain and a dimerization domain (DD), which works to increase the affinity of clients.^19^, ^2^ Ydj1 is a class 1 J-protein, and its domains can be seen in Fig. 1. Alphafold was used to show the structure of Ydj1 (UniProt identifier: A0A816B2×6), as there is not a fully elucidated crystal structure available yet (Fig. 1B).^20^, ^21^ **Class II** J-domain proteins are similar in structure; they have an N-terminal J-domain followed by the G/F-rich region. However, class II J-domain proteins lack a zinc finger domain and contain a glycine/methionine-rich region instead.^19^ All other proteins with structures that did not fit into class I or II were categorized as **class III** J-domain proteins^2^.

J-proteins work to drive the versatility of Hsp70s and their machinery, and each Hsp70 regardless of subcellular location is accompanied by its own suite of J-proteins.^2^ Humans have 11 Hsp70s but over 40 different Hsp40 proteins distributed throughout the cell.^22^ This occurrence of over 50 Hsp40s, along with their structural differences and sequence divergence, suggests they play a significant role in the versatility of Hsp70s.^2^, ^23^ Depending on their structural similarities, they are often divided into different families or types. **Type A** proteins are the closest relatives to *E. coli* DNAJ. Type A DNAJ proteins contain a J-domain (located at the N-terminal), a glycine/phenylalanine-rich region, a cysteine-rich area, and a C-terminal domain that varies.^24^ The **type B** DNAJ proteins lack the cysteine-rich region, and the **type C** DNAJ proteins only contain a J-domain, though they can be found anywhere within the protein.^24^ DNAJ proteins can dimerize, and DNAJA proteins form a dimer in the shape of a V. Hsp70 will bind to the top of the V-like structure^24^.

## The post-translational modifications of Ydj1/DNAJA1

**Fig. 2:**
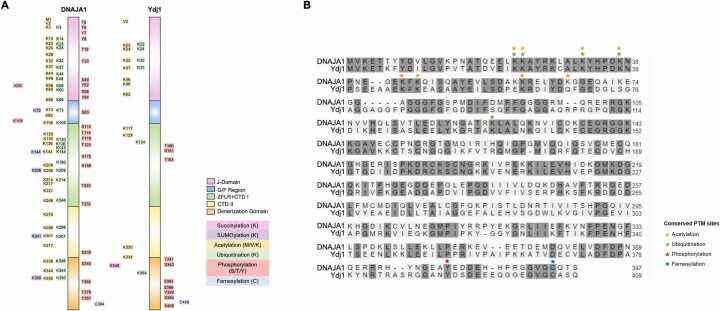
The post-translational modifications of DNAJA1 and its yeast homolog Ydj1. (A) Schematic of DNAJA1 and Ydj1 with PTM sites labeled. Data was obtained from the GPM database (https://gpmdb.thegpm.org/). (B) Amino acid alignment of DNAJA1 and Ydj1. Identical amino acids are labeled in gray. Conserved PTM sites between the two proteins are labeled with asterisks, colored based on PTM type.

**Fig. 3:**
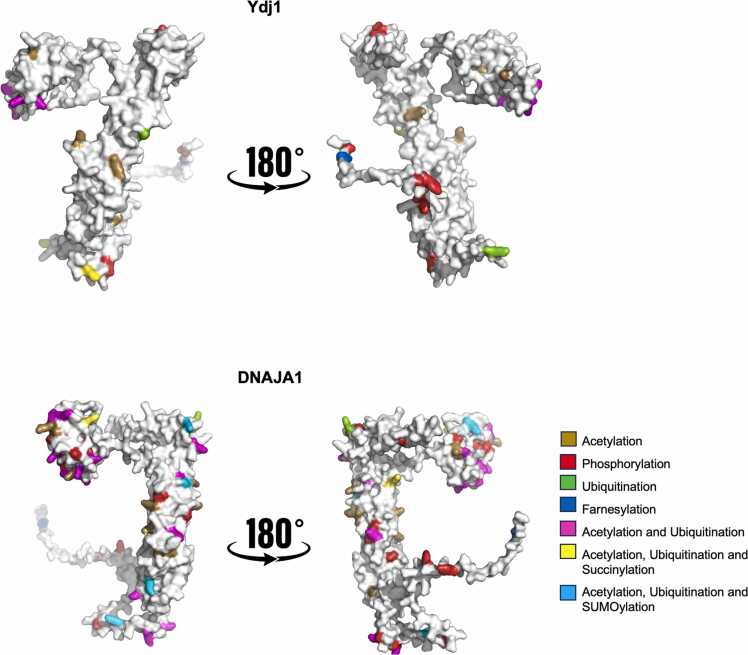
Each of the identified PTMs were mapped onto Ydj1 (AF-A0A816B2×6) and DNAJA1 (AF-A0A816B2×6-F1) predicted surface structures.

DNAJA1 and Ydj1 have five distinct regions: J-domain, glycine/phenylalanine (G/F) rich region, a carboxyl-terminal domain I (CTD I) with zinc finger-like region (ZFLD), CTD II domain, and the dimerization domain (DD).^19^ Improved proteomic technologies have led to the discovery of hundreds of post-translational modifications (PTMs) on chaperones, collectively known as the Chaperone Code.^25^, ^26^ These PTMs are thought to selectively fine-tune various molecular functions by altering activity and protein interactions. Although substantial research on the PTMs of chaperones is ongoing, the importance of Ydj1/DNAJA1 PTMs has yet to be fully explored. Ninety-two and thirty PTMs have been uncovered on DNAJA1 and Ydj1, respectively (Fig. 2A, Table 1).^27^ Despite the high conservation of amino acid sequence between Ydj1 and DNAJA1, the number of identified and conserved PTM sites between these proteins is realtivelt low (Fig. 2B). These modifications include phosphorylation, acetylation, succinylation, ubiquitination, SUMOylation, and farnesylation (Fig. 2, Fig. 3). Below is a summary of the current understanding of the regulation and potential role of PTMs on Ydj1 and DNAJA1.

**Table 1:** Distribution of PTMs per domain of DNAJA1 and Ydj1.

	**DNAJA1**					
	**J-Domain**	**G/F Region**	**ZFLR+ CTD I**	**CTD II**	**DD**	**Total Sites/ PTM**

**Acetylation**	14	3	10	4	3	34
**Phosphorylation**	10	1	8	1	4	24
**Succinylation**	1	1	0	0	0	2
**Ubiquitination**	11	2	8	2	3	26
**SUMOylation**	0	1	2	1	1	5
**Farnesylation**	0	0	0	0	1	1

**Total Sites/ Domain**	36	8	28	8	12	**92**

	**Ydj1**					
	**J-Domain**	**G/F Region**	**ZFLR+ CTD I**	**CTD II**	**DD**	**Total Sites/ PTM**

**Acetylation**	8	0	2	2	0	12
**Phosphorylation**	0	0	3	0	7	10
**Succinylation**	0	0	0	0	1	1
**Ubiquitination**	4	0	1	0	1	6
**SUMOylation**	0	0	0	0	0	0
**Farnesylation**	0	0	0	0	1	1
**Total sites/ Domain**	12	0	6	2	10	**30**

## PTMs on the J-domain

The J-domain gets its name from its predecessor, the *E. coli* protein DnaJ. It contains a short tripeptide region composed of histidine, proline, and aspartic acid called the HPD region. The J-domain helps regulate the switch from Hsp70-ATP to Hsp70-ADP, increasing the ability of Hsp70 to bind and fold proteins efficiently. While the other regions of J-proteins vary widely, the J-domain is highly conserved (Fig. 2), and even a single mutation in the HPD region can disrupt Hsp70-Ydj1/DNAJA1 interaction.^2^, ^19^ The J-domains of Ydj1/DNAJA1 are highly modified (36 PTMs on DNAJA1 and 12 PTMs on Ydj1, respectively.^27^ Interestingly, the 1st methionine on DNAJA1 and the second valine of both Ydj1 and DNAJA1 are acetylated. N-terminal acetylation typically occurs co-translationally and likely aids in the folding and initial stability of these J-proteins^28^.

Ydj1 has six additional acetylation sites on the J-domain, four of which are also ubiquitination sites (K23, K24, K32, and K34). Interestingly, all four sites are entirely conserved in the human homolog.^27^ DNAJA1s J-domain has many modification sites, including fourteen acetylation, ten phosphorylation, eleven ubiquitination, and one succinylation site.^27^ Each of the ubiquitination and succinylation sites are poly-modified.^27^ While the function of these N-terminal modifications remains unknown, given their location, it is highly likely that they mediate the J-protein-Hsp70 interaction in response to various cellular stresses. Notably, a precedent for mediation of J-protein-Hsp70 interaction by PTMs has previously been established on the chaperone side of the interaction. Phosphorylation of Hsp70 at T36 (yeast) or T38 (human) promotes dissociation of Ydj1/DNAJA1 from Hsp70, allowing the Cln3/Cyclin D1 protein to bind Hsp70 resulting in cell cycle arrest^29^.

## PTMs on the Glycine/Phenylalanine (G/F)-rich region

The Glycine/Phenylalanine (G/F) rich region links the N and C terminal domains and uniquely determines client specificity. In contrast to the J-domain, which is highly conserved, the G/F region has much more diversity, which is specific to individual J-proteins, allowing them to bind to unique clients.^30^ In mammals, mutations within the G/F region of DNAJB6 are shown to lead to an increase of cytoplasmic aggregates, which in turn causes muscle degeneration.^31^, ^32^ The G/F region is relatively small, consisting of 38 amino acid residues.^27^ There have not been any PTM sites identified in this region of YDJ1. Still, seven have been indicated in DNAJA1: three acetylation, one phosphorylation, two ubiquitination, and one SUMO.^27^ Any modification within this flexible region will likely affect the protein's shape/conformation and client specificity.

## PTMs on the carboxy-terminal domain I (CTD I)

The Carboxy-terminal domain I (CTD I) contains a hydrophobic pocket, which serves as the key client binding site. The CTD I prefers clients with hydrophobic residues that lack aromatic rings.^23^ If the CTD I binding pocket is mutated, there is a decreased ability to overcome aggregation and a loss of binding, client specificity, and refolding capabilities in conjunction with Hsp70.^33^, ^34^ While DNAJA1 boasts twenty-seven modification sites within the CTD I, five of which can be poly-modified, Ydj1 only contains six PTM sites, with no poly-mods.^27^ We would expect that any modification in this region likely plays a role in clients' correct binding and folding. PTMs in this region may be important for pairing Hsp70 paralogs with specific J-proteins.

### The Zinc finger-like domain (ZFLD)

While not all CTD I regions contain a Zinc finger-like domain, the ZFLD has recently been found to be important in peptide binding and supports the holdase activity of Ydj1.^33^ It works in cooperation with the J-domain and both CTDs to streamline the stabilization of substrates. When the ZFLR is mutated, Hsp70 luciferase refolding is diminished.^33^ If the Zinc binding motifs are deleted, there is a loss of Ydj1-Hsp70 refolding capabilities, indicating its role in suppressing aggregation.^33^ Unsurprisingly, the ZFLR is one of the most highly acetylated regions of these co-chaperones, as many lysine deacetylases (KDACs) are Zn2 + dependent.^27^, ^35^ Acetylation in this domain likely affects client binding and specificity carried out in the ZFLR and CTD regions.

## Carboxy-terminal domain II (CTD II)

The Carboxy-terminal domain II (CTD II) domain contains a hydrophobic pocket similar to the one found in CTD I; however, it is wider and typically prefers to interact with sequences containing negatively charged residues, followed by an aromatic ring.^23^ The tail of the Dimerization Domain wraps around the CTD II, leading to partial auto-inhibition, which can be released upon client binding.^23^, ^33^ This is also where the carboxy-termini of Hsp70 have been shown to bind to or associate with the Hsp40 protein.^23^ Strikingly, this is the region with the least identified number of phosphorylation sites in both the human and yeast homologs. There is a single phosphorylation site on DNAJA1, and no phosphorylation occurs on Ydj1 within this domain; the only PTM found on Ydj1s CTD II is acetylation at K320 and K334. This may indicate that this area of the protein does not require the same dynamic regulation as other portions of the protein, or stearic hindrance from the hydrophobic pocket may prevent their modification.

## Dimerization domain (DD)

The Dimerization Domain (DD) is only 72 amino acids long, yet it includes five types of PTMs: acetylation, ubiquitination, phosphorylation, SUMOylation, and farnesylation. This region is critical for Ydj1/DNAJA1 dimerization. It is positioned alongside the CTD II and allows for covalent homodimers to be formed between J-proteins.^23^ This interaction occurs when the tails wrap around the CTD II and create a hydrophobic pocket where the two proteins are bound together, allowing autoinhibition.^23^ Interestingly, phosphorylation sites are only found in the yeast homolog's CTD I and dimerization domain, indicating that these sites may be responsible for the regulation of not only client binding but the dimeric binding to and autoinhibition of Ydj1, as its negative charge will lend to creating the hydrophobic pocket that is seen when the DD tails wrap around the CTD. This idea is further supported by the fact that phosphorylation has also been seen to be important in the homodimerization of other chaperone proteins.^36^ While sumoylation is not found in Ydj1, there is a single SUMO-site (K350) on DNAJA1. This may indicate that higher-order organisms such as humans have developed a more robust regulation of this region over time through poly-modification.

Farnesylation is the most studied and well-defined PTM on Ydj1/DNAJA1. A single farnesylation site is located in the dimerization domain of Ydj1 and DNAJA1. Ram1-mediated farnesylation of Ydj1 at C406 facilitates the localization of Ydj1 to the outside of the endoplasmic reticulum (ER).^3^, ^37^, ^38^ This is particularly important, as Ydj1 is a vital ER-associated degradation (ERAD) pathway member, which tags proteins for degradation by facilitating ubiquitination.^39^, ^40^ One of these proteins is Ste6, whose human homolog (ABCB1) is responsible for multi-chemotherapeutic drug resistance.^39^, ^40^ More recently, studies have also demonstrated a role for Ydj1 in confining age-dependent aggregation deposits to mother cells.^41^ This process is dependent on Ydj1 farnesylation, which keeps the aggregates anchored to the ER of the aging cell.^38^ Confusingly, although the majority of Ydj1 is farnesylated, its localization is overwhelmingly cytosolic.^42^ This may suggest that the ER-membrane localization of Ydj1 is a highly dynamic process or that other PTMs may work with C406 farnesylation to localize Ydj1. Ablation of Ydj1 farnesylation appears to inhibit only a subset of its functions. While Ydj1-C406S yeast are unable to grow at elevated temperatures or in the presence of the replicative stress agent hydroxyurea, they present as wild-type cells in unstressed conditions.^5^, ^13^ It is also apparent that while farnesylation is needed for the activity and stability of a variety of client proteins, interactions between the client and Ydj1, Hsp90, and Hsp70 are changed in more subtle ways. In Ydj1-C406S cells, the interaction between the Ste11 kinase and Hsp70, Hsp90, and Ydj1 is substantially diminished. In contrast, while glucocorticoid receptor (GR) interaction with Hsp90 and Ydj1 is prevented in C406S cells, the interaction between GR and Hsp70 is maintained.^38^ Although more data is needed, taken together, these results seem to suggest that farnesylation of Ydj1 may be necessary for the transfer of a subset of client proteins from Hsp70 to Hsp90.

In mammalian cells, the farnesylation of DNAJA1 is also vital for client-protein interactions. Previous studies identified p53, an important tumor suppressor, as a bona fide client of the Hsp70 system.^43^, ^44^, ^45^ Mutation of the DNAJA1 farnesylation site renders p53 unstable and initially suggested that preventing DNAJA1 farnesylation may be a viable anticancer strategy.^46^ Interestingly, this theory has been given weight by recent studies that revealed a commonly used cholesterol-lowering drug, atorvastatin, can inhibit p53-driven pancreatic cancer.^47^ The mechanism of action appears to be through the inhibition of DNAJA1 farnesylation and destruction of mutant p53.^46^, ^48^ Given the challenges of inhibiting chaperones in cancer, manipulating co-chaperone PTMs may offer novel therapeutic strategies to investigate.

## Future perspectives

**Fig. 4:**
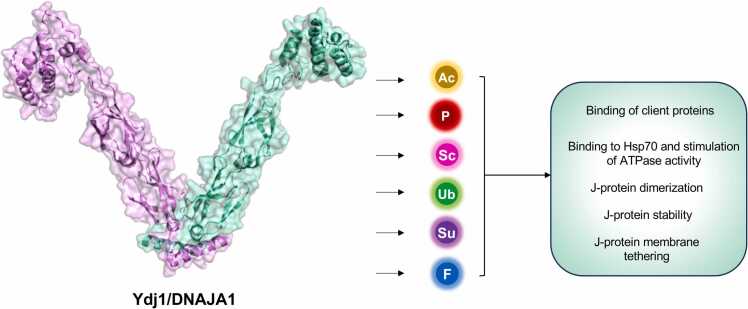
Summary of the potential roles for post-translational modifications of Ydj1 and DNAJA1.

Although substantial effort has been put into understanding the PTMs of Hsp70 and Hsp90, there is currently little understanding of the impact of PTMs on J-proteins, especially Ydj1 and DNAJA1.^25^, ^26^, ^49^ Given the location of identified PTMs on Ydj1 and DNAJA1, it is likely that they would have a major impact on co-chaperone function. This may include the initial binding of client proteins, subsequent attachment to Hsp70, and stimulation of ATPase activity, as well as the localization of the co-chaperones in the cell (Fig. 4). Several important questions remain, including the distribution of PTMs throughout the domains of Ydj1/DNAJA1. It may be that the regions on these proteins that are heavily modified are critical for co-chaperone activity, or it may be that not all PTMs on the other domains have been uncovered yet. While PTMs are dynamic, often being regulated by cellular stresses, most proteomic data for these proteins has been performed on cells in unstressed conditions. In addition, it is important to remember that not all PTMs are easily detectable by standard mass spectrometry due to either their stability or the properties of PTM-modified peptides when digested with trypsin. Future research will undoubtedly focus on the role and regulation of Ydj1/DNAJA1 sites. Of particular interest will be sites that are modified by multiple PTMs. These PTMs would be mutually exclusive and may indicate residues that act as a crossroads in the fine-tuning of co-chaperone function. We also imagine research expanding to encompass cross-talk between PTMs on chaperones and co-chaperones. For example, it is possible that PTMs on Hsp70 and DNAJA1/Ydj1 that alter their interaction are co-regulated. Alternatively, these modifications can be activated by distinct cellular pathways to allow a much finer control of the proteostasis network than previously anticipated.

## Declaration of Competing Interest

The authors declare the following financial interests/personal relationships which may be considered as potential competing interests: Andrew Truman reports financial support was provided by National Institutes of Health. Megan Mitchem reports financial support was provided by National Science Foundation.
